# Phase 2 study of lenvatinib monotherapy as second-line treatment in unresectable biliary tract cancer: primary analysis results

**DOI:** 10.1186/s12885-020-07365-4

**Published:** 2020-11-16

**Authors:** Makoto Ueno, Masafumi Ikeda, Takashi Sasaki, Fumio Nagashima, Nobumasa Mizuno, Satoshi Shimizu, Hiroki Ikezawa, Nozomi Hayata, Ryo Nakajima, Chigusa Morizane

**Affiliations:** 1 Kanagawa Cancer Centre Hospital, Yokohama, Japan; 2 Department of Hepatobiliary and Pancreatic Oncology, National Cancer Center Hospital East, 6-5-1 Kashiwanoha, Kashiwa, 277-8577 Japan; 3 Cancer Institute Hospital of JFCR, Tokyo, Japan; 4 Kyorin University, Tokyo, Japan; 5 Aichi Cancer Centre Hospital, Nagoya, Japan; 6 Saitama Cancer Centre, Saitama, Japan; 7 Eisai Co. Ltd., Tokyo, Japan; 8 Eisai Inc., Woodcliff Lake, NJ USA; 9 National Cancer Centre Hospital, Tokyo, Japan

**Keywords:** Lenvatinib, Biliary tract cancer, Cholangiocarcinoma, Gallbladder cancer, Ampulla of Vater

## Abstract

**Background:**

Biliary tract cancer (BTC) has a poor prognosis and lacks a standardized second-line therapy. Vascular endothelial growth factor (VEGF), fibroblast growth factor receptor (FGFR) 4, and platelet-derived growth factor receptor (PDGFR) are highly expressed in BTC. Therefore, lenvatinib (a known inhibitor of VEGF receptors 1–3, FGFRs 1–4, and PDGFR-α) was evaluated for second-line treatment of BTC.

**Methods:**

In this single-arm, multicenter, open-label, phase 2 study, patients with BTC received lenvatinib 24 mg orally once daily in 28-day cycles. The primary endpoint was objective response rate (ORR). Secondary endpoints included overall survival (OS), progression-free survival (PFS), PFS rate at 12 weeks, disease control rate, clinical benefit rate, safety and pharmacokinetic profiles.

**Results:**

Twenty-six Japanese patients were enrolled and treated; 3 had a confirmed partial response per investigator assessment and per independent imaging review (IIR); ORR was 11.5% (90% confidence interval [CI]: 3.2–27.2). Median PFS was 3.19 months (95% CI: 2.79–7.23) per investigator assessment and 1.64 months (95% CI: 1.41–3.19) per IIR. Median OS was 7.35 months (95% CI: 4.50–11.27). Grade ≥ 3 treatment-emergent adverse events (TEAEs) occurred in 21 patients (80.8%) and included hypertension (*n* = 10 [38.5%]), proteinuria (*n* = 3 [11.5%]), palmar-plantar erythrodysesthesia (n = 3 [11.5%]), decreased appetite (n = 3 [11.5%]), and anemia (n = 3 [11.5%]). Two deaths occurred due to TEAEs between treatment initiation and 30 days after last dose, but neither were considered treatment related.

**Conclusions:**

Lenvatinib demonstrated antitumor activity in BTC, with a tolerable safety profile, and should be further evaluated as potential second-line therapy for this difficult to treat population.

**Trial registration:**

ClinicalTrials.gov NCT02579616. Date of registration: October 19, 2015.

## Background

Biliary tract cancer (BTC) is the second-most-common hepatobiliary cancer worldwide [1, 2] and includes gallbladder cancer, intrahepatic cholangiocarcinoma, and extrahepatic cholangiocarcinoma [3]. Ampulla of Vater cancer is sometimes characterized as a biliary tract cancer [1, 3], although the National Comprehensive Cancer Network does not include it under hepatobiliary cancers [4]. BTC incidence increased 25% worldwide between 2007 and 2017 (approximately 174,000 deaths) according to a Global Burden of Disease study [5]. A recent US study reported increasing incidence rates of gallbladder cancer in younger patients (< 45 years of age; 1.8% increase per year) and African Americans [3]. Patients with BTC have a poor prognosis and a shortened life expectancy (typically ≤1 year following diagnosis) [1, 6].

Currently, radical surgery is the only potentially curative therapy, but this is not an option for many patients who present with advanced disease [7]. The standard first-line therapy for BTC is gemcitabine and cisplatin (GC) [4]. Gemcitabine plus S-1 (GS) [8] and GC plus S-1 have demonstrated potential as first-line therapies [9]. Unfortunately, for patients who progress on or after first-line therapies there are no approved subsequent treatment options [4]. Thus, there is an unmet need for an effective second-line therapy for patients with BTC.

Vascular endothelial growth factor (VEGF), fibroblast growth factor receptor (FGFR) 4, and platelet-derived growth factor receptor (PDGFR) are highly expressed in patients with BTC and correlate with a poor prognosis [10–13]. Lenvatinib is an oral multikinase inhibitor that targets VEGF receptors 1–3, FGFRs 1–4, PDGFRα, RET, and KIT [14–17]. Lenvatinib monotherapy is approved for the treatment of radioiodine-refractory differentiated thyroid cancer (DTC) and first-line treatment of hepatocellular carcinoma in Japan, the United States, Europe, China, and several other countries [18].

The overexpression of VEGF, FGFR, and PDGFR in BTC suggests that lenvatinib could play a role in the treatment of BTC. This phase 2 study evaluated the safety and efficacy of lenvatinib as second-line therapy for patients with BTC [19].

## Methods

### Study design

This study (Study 215; NCT02579616) was a single-arm, multicenter, open-label, phase 2 study in Japanese patients with unresectable BTC. Patients received lenvatinib 24 mg orally once daily in 28-day cycles. Treatment continued until development of an unacceptable toxicity, disease progression, withdrawal of consent, or documentation of significant violations of the prespecified inclusion/exclusion criteria.

The primary endpoint was objective response rate (ORR). Secondary endpoints included overall survival (OS), progression-free survival (PFS), PFS rate at 12 weeks, disease control rate (DCR), clinical benefit rate (CBR; the proportion of patients with complete response + partial response + durable stable disease [≥ 23 weeks]), and safety and pharmacokinetic profiles. Tumor assessments were performed every 6 weeks until week 24, and then every 8 weeks thereafter, utilizing Response Evaluation Criteria In Solid Tumors (RECIST), version 1.1, by investigator assessment for the primary analysis. Independent imaging review (IIR) was utilized to support the post hoc analysis. Complete or partial responses required confirmation ≥28 days after the initial response.

The safety profile was assessed by monitoring and recording all adverse events (AEs), including all Common Terminology Criteria for Adverse Events, version 4.03, grades and serious AEs; periodic laboratory evaluations for hematology, blood chemistry, and urine values; periodic measurement of vital signs; electrocardiograms; and physical examinations. Toxicity was managed by supportive medications, treatment interruption, dose reduction (to 20 mg, 14 mg, or 10 mg; re-escalation was not allowed), and/or treatment discontinuation in accordance with protocol-prespecified dose-modification guidelines. Briefly, hypertension was managed by initiating antihypertensives if blood pressure was ≥ 140 mmHg (systolic) or ≥ 90 mmHg (diastolic), and then by dose interruption and reduction if blood pressure was ≥ 160 mmHg (systolic) or ≥ 90 mmHg (diastolic), despite optimal management with antihypertensive medications. Lenvatinib was discontinued upon occurrence of any grade ≥ 4 treatment-related AEs.

Plasma samples were collected from all patients on cycle 1, day 1 (C1D1; postdose), C1D8 (predose), C1D15 (pre/postdose), and C2D1 (predose) to assess the pharmacokinetic profile. Validated liquid chromatography with tandem mass spectrometry was utilized to determine lenvatinib plasma concentrations. Plasma concentrations were compared to the levels observed in patients from Study 303 (a phase 3 study of patients with DTC treated with lenvatinib 24 mg once daily) [20].

The primary analysis was performed as planned at the data cut-off (November 22, 2016) when all patients had finished their week 32 tumor assessment or had discontinued treatment. All patients provided written informed consent. The study protocol, informed consent form and any related documents were submitted to an Institutional Review Board for approval. This study was conducted in accordance with the World Medical Association Declaration of Helsinki, Good Clinical Practices, and local ethical/legal requirements.

### Eligibility

Patients enrolled must have experienced disease progression or treatment failure following 1 prior gemcitabine-based chemotherapy regimen (in combination with cisplatin or other platinum agent/fluoropyrimidine agent). Pathologically or cytologically confirmed unresectable adenocarcinoma of BTC, measurable disease per RECIST version 1.1, and an Eastern Cooperative Oncology Group performance status (ECOG PS) score of 0 or 1 were required. Additionally, eligible patients were required to be ≥ 20 years old with adequately controlled blood pressure (≤ 150/90 mmHg), adequate blood coagulation, and major organ function.

Key exclusion criteria comprised: any anticancer treatment within 21 days prior to the first dose of study drug, bleeding/thrombotic disorders, meningeal carcinomatosis, unstable brain/subdural metastases, or New York Heart Association Class ≥ 2 heart failure.

### Statistical analysis

The sample size was determined based on the width of confidence interval (CI) using the 1-sample binomial distribution. Approximately, 25 patients were to be enrolled and if the true ORR was 15%, 3 (90% CI: 3.4–28.2) to 4 (90% CI: 5.7–33.0) responses were expected. Efficacy and safety, and pharmacokinetic assessments were performed on all patients who received at least 1 dose of study drug. The ORR, DCR, CBR, and corresponding exact 2-sided 90% CIs, were calculated using the Clopper–Pearson method. These endpoints were evaluated by investigator assessment and IIR. The Kaplan–Meier method was utilized to summarize OS, PFS, and PFS rate at 12 weeks. The Greenwood formula and log-log transformation were used to calculate the 95% CIs.

## Results

### Patients

This study enrolled 26 Japanese patients, and all patients received at least 1 dose of lenvatinib. Primary tumor locations included gallbladder (*n* = 10), extrahepatic bile duct (*n* = 8), intrahepatic bile duct (*n* = 6), and the ampulla of Vater (*n* = 2). Most patients were male (57.7%), had an ECOG PS score of 0 (73.1%), and had metastases of the lymph nodes (61.5%) or liver (57.7%) (Table 1). Additionally, the following baseline characteristics have previously demonstrated correlations with OS and/or PFS, and are shown in Table 1 according to the cutoff values used in previous research: white blood cell count, hemoglobin, alkaline phosphatase, albumin, and lesion size [7, 21].

**Table 1:** Baseline Characteristics

Category	Patients Treated With Lenvatinib 24 mg Once Daily (***N*** = 26)
**Median age, years (range)**	64 (41–78)
**Age group, n (%)**	
< 65 years	14 (53.8)
≥ 65 years	12 (46.2)
**Median weight, kg (range)**	56.9 (41.5–77.8)
**Sex, n (%)**	
Male	15 (57.7)
Female	11 (42.3)
**ECOG PS, n (%)**	
0	19 (73.1)
1	7 (26.9)
**Primary tumor location, n (%)**	
Intrahepatic bile duct	6 (23.1)
Extrahepatic bile duct	8 (30.8)
Perihilar	1 (3.8)
Distal	7 (26.9)
Gallbladder	10 (38.5)
Ampulla of Vater	2 (7.7)
**Tumor lesions at screening, n (%)**	
Adrenal	2 (7.7)
Ascites	2 (7.7)
Bile duct^a^	4 (15.4)
Gallbladder^a^	8 (30.8)
Bone	1 (3.8)
Breast	1 (3.8)
Liver	15 (57.7)
Lung	5 (19.2)
Lymph node	16 (61.5)
Peritoneal	6 (23.1)
**Lesion size**^**b**^**, n (%)**	
< 20 mm	1 (3.8)
≥ 20 mm	25 (96.2)
**Median lesion size**^**b**^**, mm (range)**	35 (16–117)
**Tumor marker (CA 19–9), median U/mL (range)**	175.2 (0.6–105,050.1)
**Tumor marker (CA 19–9), n (%)**	
≤ 152 U/mL	12 (46.2)
> 152 U/mL	14 (53.8)
**White blood cell count**	
Median, /mm^3^ (range)	5465 (3340–8900)
≤ 10,000/mm^3^, n (%)	26 (100.0)
**Hemoglobin**	
Median, g/dL (range)	12.05 (9.4–15)
≤ 12 g/dL, n (%)	13 (50.0)
> 12 g/dL, n (%)	13 (50.0)
**Total bilirubin**	
Median, mg/dL (range)	0.6 (0.3–1.2)
≤ 0.66 mg/dL, n (%)	16 (61.5)
> 0.66 mg/dL, n (%)	10 (38.5)
**Alkaline phosphatase**	
Median, U/L (range)	321 (136–1235)
≤ 247 U/L, n (%)	6 (23.1)
> 247 U/L, n (%)	20 (76.9)
**Albumin**	
Median, g/dL (range)	4.1 (2.7–4.7)
≤ 3.56 g/dL, n (%)	1 (3.8)
> 3.56 g/dL, n (%)	25 (96.2)
**Previous anticancer surgery, n (%)**	
No	20 (76.9)
Yes	6 (23.1)
**Prior chemotherapy to biliary tract cancer, n (%)**	
Adjuvant	2 (7.7)
S-1^c^	1 (3.8)
Gemcitabine	1 (3.8)
Therapeutic	26 (100.0)
Gemcitabine + cisplatin	20 (76.9)
Gemcitabine + S-1^c^	6 (23.1)
**Duration of the previous gemcitabine-based combination chemotherapy, n (%)**	
< 6 months	13 (50.0)
≥ 6 months	13 (50.0)

*CA* cancer antigen, *ECOG PS* Eastern Cooperative Oncology Group performance status

^a^14 Patients did not have lesions at the bile duct or gallbladder upon screening

^b^Per investigator assessment

^c^Combination treatment consisting of tegafur, gimeracil, and oteracil

### Efficacy

**Fig. 1:**
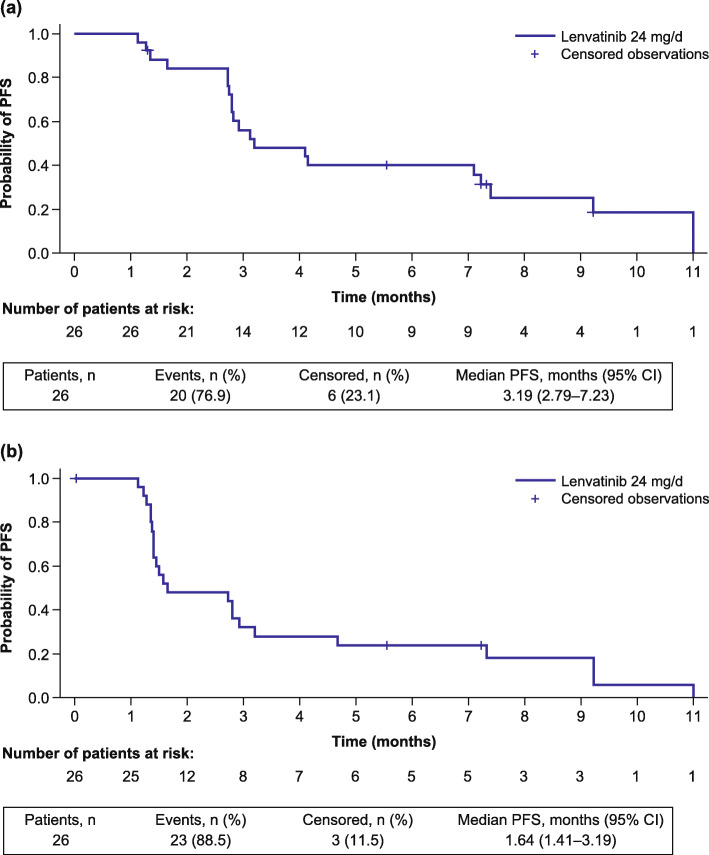
Kaplan–Meier plot of PFS by investigator assessment (**a**) and IIR (**b**). *CI*, confidence interval; *IIR*, independent imaging review; *PFS*, progression-free survival

**Fig. 2:**
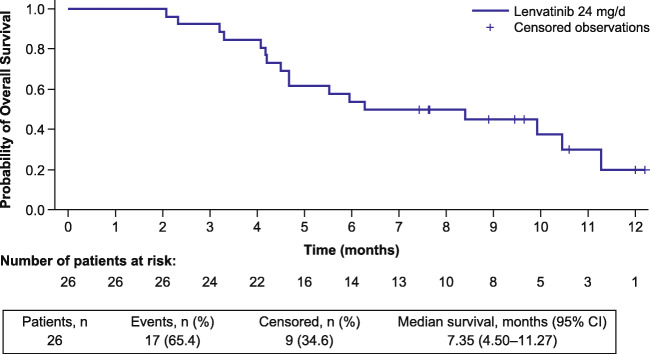
Kaplan–Meier plot of OS. ^a^17 Deaths occurred in this study; 2 deaths occurred within 30 days of administration of the last dose and 15 deaths occurred > 30 days after administration of the last dose. *CI*, confidence interval; *OS*, overall survival

**Fig. 3:**
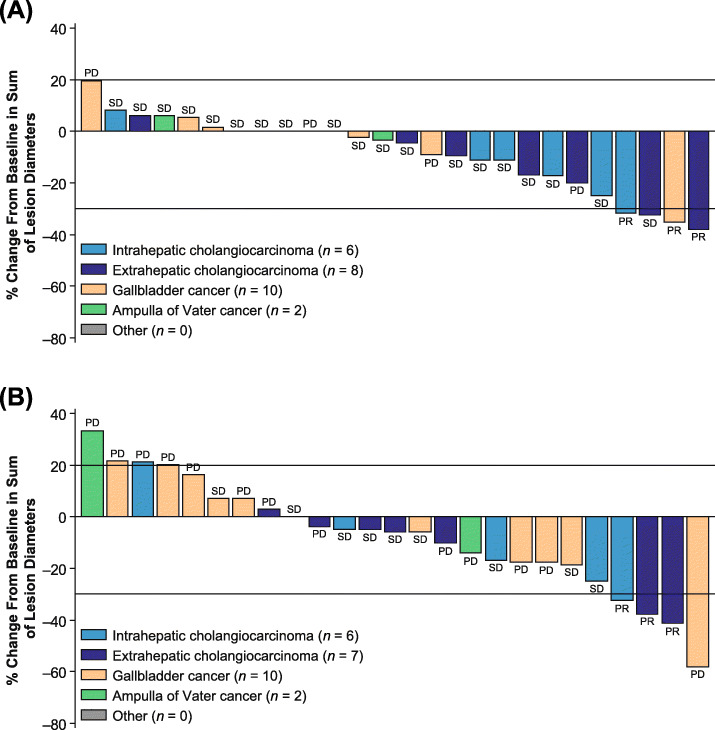
Percentage change from baseline in the sum of lesion diameters per investigator assessment (**A**) and IIR (**B**). ^a^One patient was assigned a BOR of “unknown” by IIR and was excluded from this analysis. BOR, best overall response; IIR, independent imaging review; PD, progressive disease; PR, partial response; SD, stable disease

The ORR following lenvatinib treatment was 11.5% (90% CI: 3.2–27.2) per investigator assessment; 3 patients (11.5%) experienced a partial response, and 19 patients (73.1%) achieved stable disease (Table 2). The median PFS was 3.19 months (95% CI: 2.79–7.23) (Fig. 1), and the PFS rate at 12 weeks was 72.2% (95% CI 50.4–85.7) per investigator assessment. The median OS was 7.35 months (95% CI: 4.50–11.27; Fig. 2), and most patients experienced a reduction in tumor size (Fig. 3).

**Table 2:** Efficacy Outcomes

Category	Patients Treated With Lenvatinib 24 mg Once Daily (***N*** = 26)	
	Investigator Assessment	IIR
**Objective response rate, n (%)** **(90% CI)**	3 (11.5) (3.2–27.2)	3 (11.5) (3.2–27.2)
**Best overall response, n (%)**		
Complete response	0	0
Partial response	3 (11.5)	3 (11.5)
Stable disease	19 (73.1)	9 (34.6)
Progressive disease	4 (15.4)	13 (50.0)
Not evaluable	0	0
Unknown	0	1 (3.8)
**Disease control rate**^**a**^**, n (%)** **(90% CI)**	22 (84.6) (68.2–94.6)	12 (46.2) (29.2–63.8)
**Clinical benefit rate**^**b**^**, n (%)** **(90% CI)**	10 (38.5) (22.6–56.4)	6 (23.1) (10.6–40.5)
**PFS rate at 12 weeks, % (95% CI)**	72.2 (50.4–85.7)	44.0 (24.5–61.9)
**Median PFS, months (95% CI)**	3.19 (2.79–7.23)	1.64 (1.41–3.19)
**Median time to progression, months (95% CI)**	4.11 (2.76–7.39)	1.64 (1.41–2.92)
**Median overall survival, months (95% CI)**	7.35 (4.50–11.27)	

*CI* confidence interval, *IIR* independent imaging review, *PFS* progression-free survival

^a^The proportion of patients with a best overall response of complete response, partial response or stable disease; stable disease needed to be achieved at cycle 2 day 8 or later

^b^The proportion of patients with complete response + partial response + durable stable disease (≥ 23 weeks)

Additionally, the ORR per IIR was also 11.5% (90% CI: 3.2–27.2); 3 patients (11.5%) experienced a partial response, and 9 patients (34.6%) achieved stable disease (Table 2). The median PFS was 1.64 (95% CI: 1.41–3.19) months, and the PFS rate at 12 weeks was 44.0% (95% CI: 24.5–61.9), both per IIR (Fig. 1). The DCR and CBR results per investigator assessment and IIR are shown in Table 2.

### Safety

Treatment-emergent adverse events (TEAEs) are shown in Table 3; treatment-related AEs (Table 4) occurred in all 26 patients. The most common TEAEs (occurring in ≥ 50% of patients) were hypertension (84.6%), dysphonia (61.5%), proteinuria (61.5%), palmar-plantar erythrodysesthesia syndrome (57.7%), decreased appetite (53.8%), thrombocytopenia (53.8%), and fatigue (50%) (Table 3). TEAEs of grade ≥ 3 severity were reported in 21 patients. Two deaths occurred due to TEAEs (cholangitis, *n* = 1; completed suicide, *n* = 1) between the initiation of treatment and 30 days from the last dose, but neither were considered related to treatment by the investigators.

**Table 3:** Treatment-emergent Adverse Events That Occurred in ≥10% of Patients

TEAE^**a**^	Patients Treated With Lenvatinib 24 mg Once Daily (***N*** = 26)	
**Patients with any-grade TEAE, n (%)**	26 (100)	
**Patients with any TEAE ≥ grade 3, n (%)**	21 (80.8)	
**Preferred term, n (%)**	**Any grade**	**Grade 3 or 4**
Hypertension	22 (84.6)	10 (38.5)
Dysphonia	16 (61.5)	0
Proteinuria	16 (61.5)	3 (11.5)
Palmar-plantar erythrodysesthesia syndrome	15 (57.7)	3 (11.5)
Decreased appetite	14 (53.8)	3 (11.5)
Thrombocytopenia	14 (53.8)	1 (3.8)
Fatigue	13 (50.0)	0
Hypothyroidism	12 (46.2)	0
Peripheral edema	9 (34.6)	0
Constipation	8 (30.8)	0
Decreased weight	8 (30.8)	0
Diarrhea	8 (30.8)	1 (3.8)
Pyrexia	8 (30.8)	0
Anemia	6 (23.1)	3 (11.5)
Cholangitis	6 (23.1)	4 (15.4)
Nausea	6 (23.1)	0
Rash	6 (23.1)	1 (3.8)
Upper abdominal pain	6 (23.1)	0
Malaise	5 (19.2)	0
Ascites	4 (15.4)	2 (7.7)
Cancer pain	4 (15.4)	0
Headache	4 (15.4)	0
Myalgia	4 (15.4)	0
Stomatitis	4 (15.4)	0
Tumor pain	4 (15.4)	0
Alopecia	3 (11.5)	0
Bile duct obstruction	3 (11.5)	2 (7.7)
Delirium	3 (11.5)	0
Epistaxis	3 (11.5)	0
Hypoalbuminemia	3 (11.5)	2 (7.7)
Hypophosphatemia	3 (11.5)	1 (3.8)
Lymphopenia	3 (11.5)	2 (7.7)
Pruritis	3 (11.5)	0
Vomiting	3 (11.5)	0

*TEAE* treatment-emergent adverse event

^a^TEAEs were any adverse events that occurred between initiation of treatment and 30 days from last dose

**Table 4:** Treatment-related Adverse Events That Occurred in ≥10% of Patients

TRAE	Patients Treated With Lenvatinib 24 mg Once Daily (***N*** = 26)	
**Patients with any-grade TRAE, n (%)**	26 (100)	
**Patients with any TRAE ≥ grade 3, n (%)**	16 (61.5)	
**Preferred term, n (%)**	**Any grade**	**Grade 3 or 4**
Hypertension	22 (84.6)	10 (38.5)
Dysphonia	16 (61.5)	0
Palmar-plantar erythrodysesthesia syndrome	15 (57.7)	3 (11.5)
Proteinuria	15 (57.7)	3 (11.5)
Thrombocytopenia	13 (50.0)	1 (3.8)
Decreased appetite	12 (46.2)	0
Fatigue	12 (46.2)	0
Hypothyroidism	12 (46.2)	0
Decreased weight	6 (23.1)	0
Diarrhea	6 (23.1)	0
Anemia	5 (19.2)	1 (3.8)
Malaise	5 (19.2)	0
Nausea	5 (19.2)	0
Peripheral edema	5 (19.2)	0
Myalgia	4 (15.4)	0
Stomatitis	4 (15.4)	0
Alopecia	3 (11.5)	0
Constipation	3 (11.5)	0
Epistaxis	3 (11.5)	0
Headache	3 (11.5)	0
Rash	3 (11.5)	1 (3.8)
Upper abdominal pain	3 (11.5)	0

*TRAE* treatment-related adverse event

TEAEs led to treatment discontinuation in 2 patients (7.7%; erythema multiforme, *n* = 1; and lung abscess, *n* = 1). However, most TEAEs were manageable: 76.9% (20/26) of patients required lenvatinib dose reduction and 65.4% (17/26) of patients required dose interruption. The most common TEAEs leading to dose reduction were decreased appetite (6/26; 23.1%), fatigue (5/26; 19.2%), thrombocytopenia (5/26; 19.2%), proteinuria (4/26; 15.4%), and palmar-plantar erythrodysesthesia syndrome (3/26; 11.5%). The most common TEAE leading to dose interruption was cholangitis (3/16; 11.5%). Patients received a median of 4.0 cycles (range, 1 to 40 cycles) of lenvatinib; 6 patients received ≥ 10 cycles. The median duration of treatment was 3.1 months (range, 0.5 to 36.8 months).

### Pharmacokinetic profile

**Fig. 4:**
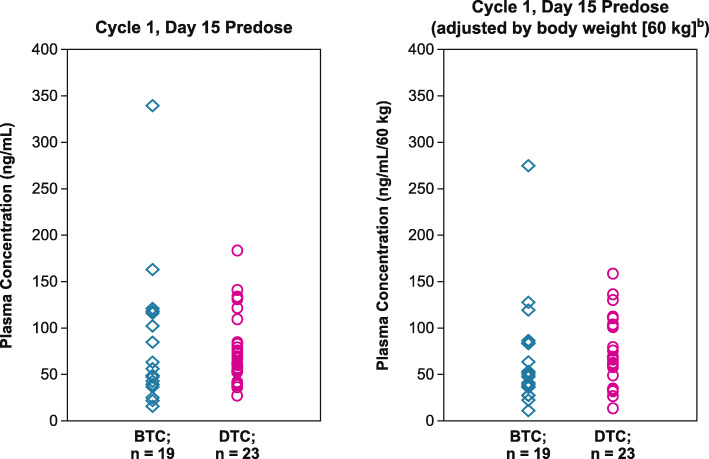
Comparison of lenvatinib plasma concentration in patients with BTC (this study) to patients with DTC (Study 303) [20]. ^a^There were 7 patients excluded from the pharmacokinetic analysis because their dose was reduced or interrupted before cycle 1 day 15. ^b^Bodyweight-adjusted plasma concentration was calculated as follows: individual plasma concentration × bodyweight [kg]/60 [kg]. *BTC*, biliary tract cancer; *DTC*, differentiated thyroid cancer

Lenvatinib trough plasma concentrations in Japanese patients with BTC were comparable to the levels seen in a previous study of Japanese patients with DTC (Study 303) [20]. Plasma concentrations and body-weight-adjusted plasma concentrations from both studies at C1D15 are shown in Fig. 4.

## Discussion

Here, we report the results of a phase 2 study evaluating lenvatinib as a second-line treatment option in patients with BTC who have failed gemcitabine-based therapy. The ORR was 11.5% (90% CI: 3.2–27.2) per investigator assessment and per IIR. There was a notable difference in the number of patients considered to have achieved stable disease between investigator assessment (*n* = 19) and IIR (*n* = 9). Potentially, this was because of the reviewers’ differing perception of the response based on RECIST version 1.1 criteria. Because numerous factors are considered in the determination of progressive versus stable disease, including both change in target and nontarget lesions, and overall tumor burden, individual reviewers may evaluate the response differently. Lenvatinib demonstrated antitumor activity with a median OS of 7.35 months (95% CI: 4.50–11.27) and a median PFS (per investigator assessment) of 3.19 months (95% CI: 2.79–7.23) (vs 1.64 months per IIR; 95% CI: 1.41–3.19).

Recent studies have investigated other second-line therapy options: one phase 3 study (NCT01926236) suggested that the modified FOLFOX (mFOLFOX) chemotherapy regimen consisting of oxaliplatin and 5-fluorouracil should be considered the default second-line treatment for advanced/metastatic BTC [22]. The results of this study, which evaluated active symptom control (ASC) versus ASC plus mFOLFOX, were presented at ASCO 2019. The ASCO presentation reported a median OS of 6.2 months [22], median PFS of 4.0 months, ORR of 5%, and DCR of 33% in the ASC plus mFOLFOX arm. A statistically significant (*P* = 0.031) and clinically meaningful improvement in OS was observed in patients treated with ASC plus mFOLFOX versus ASC alone. Another chemotherapy regimen, FOLFIRINOX, demonstrated efficacy in a phase 2 trial of patients with BTC who had experienced disease progression following treatment with cisplatin and gemcitabine: median PFS and OS were 6.2 and 10.7 months, respectively [23, 24]. Also, Abou-Alfa et al. [25] evaluated ivosidenib versus placebo in patients with advanced cholangiocarcinoma (primarily intrahepatic) and an isocitrate dehydrogenase 1 (*IDH1*) gene mutation. Of note, this population differed from our study, which enrolled patients without regard to a specific gene mutation. This phase 3 study, which allowed crossover from placebo to ivosidenib, reported the first positive PFS data of molecularly targeted therapy in cholangiocarcinoma [25]. Median PFS was 2.7 months versus 1.4 months in the ivosidenib and placebo arms, respectively (hazard ratio 0.37; 95% CI: 0.25–0.54; *P* < 0.001). Median OS was longer in the ivosidenib arm versus placebo (10.8 vs 9.7 months) but these results were not significant (*P* = 0.06) [25].

Previous studies have evaluated the efficacy of other tyrosine kinase inhibitors in the treatment of patients with advanced BTC [26–29]. A phase 2 study of regorafenib as second-line treatment in 43 patients with metastatic BTC demonstrated favorable results with 11% (*n* = 5) of patients achieving a partial response and a median PFS of 15.6 weeks (90% CI: 12.9–24.7 weeks) [27], which is approximately 3.9 months. Sunitinib demonstrated marginal efficacy as second-line treatment in a phase 2 study (*n* = 56) of metastatic BTC with a median time to progression of 1.7 months (95% CI: 1.0–2.4) and an ORR of 8.9% [28]. Lastly, a phase 2 study of sorafenib in patients with unresectable or metastatic gallbladder carcinoma and cholangiocarcinoma demonstrated a median PFS of 3 months (95% CI: 2–4) but was terminated early because it failed to meet the primary objective (ORR of 20%) [29]. However, a pilot study of sorafenib versus best supportive care in patients with advanced intrahepatic cholangiocarcinoma demonstrated sorafenib has antitumor activity with a median PFS of 3.2 months (95% CI: 2.4–4.1) and median OS of 5.7 months (95% CI: 3.7–8.5) [26]. Additionally, several phase 2 trials evaluating tyrosine kinase inhibitors, such as apatinib (NCT03521219), infigratinib (NCT02150967), derazantinib (NCT03230318), erdafitinib (NCT02699606), and pemigatinib (NCT04256980), as second-line treatment options in patients with BTC are currently ongoing [23].

In addition to direct antitumor activity, lenvatinib has also demonstrated immunomodulatory activity. Research has shown that lenvatinib modulates cancer immunity in the immunocompetent tumor microenvironment by reducing the population of tumor-associated macrophages (TAMs) and increasing the population of interferon-γ– and granzyme-B–producing CD8+ T cells [30, 31]. Further, lenvatinib demonstrated enhanced antitumor activity preclinically via the interferon-signaling pathway in combination with a programmed cell death-1 (PD-1) inhibitor [31]. Atanasov et al. [32] evaluated the relationship between the prevalence of TAMs and tumor growth in patients with hilar cholangiocarcinoma, a subtype of BTC. This study reported that overall tumor recurrence was significantly higher in patients with high levels of TAMs at the tumor invasive fronts compared with patients with low levels of TAMs (69.2% vs 33.3%; *P* = 0.015). Patients with high levels of TAMs experienced worse survival outcomes [32]. These preclinical data suggest that lenvatinib in combination with a PD-1 inhibitor may demonstrate further improved outcomes and therefore should be investigated. Further, a phase 2 trial of lenvatinib in combination with pembrolizumab or nivolumab in patients with intrahepatic cholangiocarcinoma who previously received ≥ 2 anticancer treatments, demonstrated promising results with an ORR of 21.4% and a median PFS of 5.9 months (95% CI: 4.2–6.2) [23, 33]. Of note, studies of lenvatinib in combination with PD-1 inhibitors (pembrolizumab [NCT03797326] and nivolumab [JMA-IIA00436]) in patients with BTC are currently ongoing.

## Conclusions

Lenvatinib demonstrated promising antitumor activity in patients with unresectable BTC who had failed gemcitabine-based chemotherapy. Additionally, the safety profile of lenvatinib in patients with BTC is similar to that previously reported in patients with other tumor types [34–36], and no new safety signals were identified. Toxicities were manageable with treatment modifications, dose reductions, or discontinuations. Noteworthy limitations of this study include the small sample size (*n* = 26), and the large diversity in BTC subgroups that were included (gallbladder cancer, *n* = 10; intrahepatic cholangiocarcinoma, *n* = 6; extrahepatic cholangiocarcinoma, *n* = 8; and ampulla of Vater cancer, *n* = 2). However, the inclusion of several BTC subgroups allows for easier trial enrollment, which in turn enables earlier clinical advances in the treatment of BTC. Further investigation of lenvatinib monotherapy or combination therapy with a PD-1 inhibitor as a potential second-line treatment option for patients with unresectable BTC is warranted, and ideally should be conducted in larger patient populations of each BTC subgroup.

## Data Availability

The data will not be available for sharing at this time as the data are commercially confidential. However, written requests to share the data will be considered on a case-by-case basis. To request data, please contact Dr. Masafumi Ikeda (email: masikeda@east.ncc.go.jp).

## Abbreviations

AE: Adverse event
ASC: Active symptom control
BTC: Biliary tract cancer
C#D#: Cycle #, day #
CBR: Clinical benefit rate
CI: Confidence interval
DCR: Disease control rate
DTC: Differentiated thyroid cancer
ECOG PS: Eastern Cooperative Oncology Group performance status
FGFR: Fibroblast growth factor receptor
GC: Gemcitabine and cisplatin
GS: Gemcitabine plus S-1
IIR: Independent imaging review
mFOLFOX: modified FOLFOX
ORR: Objective response rate
OS: Overall survival
PD-1: Programmed cell death-1
PDGFR: Platelet-derived growth factor receptor
PFS: Progression-free survival
RECIST: Response Evaluation Criteria In Solid Tumors
TAM: Tumor-associated macrophages
TEAE: Treatment-emergent adverse event
VEGF: Vascular endothelial growth factor
